# Paroxetin in the treatment of depressive symptoms and insomnia

**DOI:** 10.1192/j.eurpsy.2025.1325

**Published:** 2025-08-26

**Authors:** E. S. Gisbert, M. J. A. Abildua

**Affiliations:** 1 PSYCHIATRY/NEUROLOGY, HOSPITAL UNIVERSITARIO INFANTA SOFIA, SAN SEBASTIAN DE LOS REYES, Spain

## Abstract

**Introduction:**

OBJECTIVES: Insomnia is the most prevalent sleep disorder in the general population and depresive symtoms, and one of the most frequent reasons for consultation in the Sleep Units. Paroxetin is an antidepresive also effective on the structure of sleep.

**Objectives:**

We describe the study that evaluates Paroxetin in patients with depression and insomnia.

**Methods:**

Observational retrospective study of 40 patients with depression and insomnia. All patients attended in Neurology or Psychiatry Consultation, from November 2022 to November 2023. They were treated with Paroxetin 30 mgrs. We reviewed age, sex, depressive symtoms and insomnia, and the results of paroxetin for insomnia after 6 months of treatment, measured by the improvement of 3 or more points in the ISI and Pittsburgh scales, as well as the average of hours of sleep gained. The depressive symtoms measured with Hamilton scales.

**Results:**

RESULTS: 40 patients with depression and insomnia, 24 women (60%), 16 men (40%). Average age 46,5 years

Main etiology: depression 40 cases (100%). After the treatment with Paroxetin the total number of hours of sleep improves in 2.5 hours, the scale ISI improves by 6 points (± 2.1 SD, p=0.02), and Pittsburgh scale improves in 4 points (± 1.7, p =0.04). The Hamilton scales improves in 19 points. The treatment was abandoned by 2 patients (5%): 1 due to pregnancy wish and 1 due digestive symtoms.

**Conclusions:**

CONCLUSION: The Paroxetin 30 mgrs/day improves the quality of sleep measured by ISI and Pittsburgh scales (statistically significant), and improves the depressive symtoms.

**Disclosure of Interest:**

None Declared

